# Asymptomatic Appendiceal Metastasis From Prostate Cancer Mimicking Metastatic Lymph Node as Solitary Disease on PSMA PET/CT Scan: The First Reported Case of Presurgical Diagnosis and Review of Literature

**DOI:** 10.1155/criu/7673609

**Published:** 2026-10-22

**Authors:** Sunantha S. Ploysongsang, Yongyut Kongthanarat, Teerakul Jirotmontree, Sukij Panpimanmas, Vorachai Sirikulchayanonta

**Affiliations:** ^1^ Department of Radiation Oncology MedPark Hospital Bangkok Thailand; ^2^ Department of Surgery MedPark Hospital Bangkok Thailand; ^3^ Department of Pathology MedPark Hospital Bangkok Thailand; ^4^ Faculty of Science Rangsit University Pathum Thani Thailand rsu.ac.th

**Keywords:** appendix, complicated appendicitis, metastasis, prostate cancer, PSMA PET/CT scan

## Abstract

Metastasis of prostate cancer to the appendix is exceedingly rare. We can find only 11 cases reported in the world English literature. Their diagnoses were made postoperatively after presenting symptoms of appendicitis and at the time of surgery; many were found to have complicated appendicitis. We are reporting a new case of this rare disease with the diagnosis being made preoperatively, in absence of the symptoms. The follow‐up prostate‐specific membrane antigen (PSMA) PET/CT scan revealed a solitary abnormal finding at the medial right common iliac lymph node (LN) area and was interpreted as an LN metastasis. On CT simulation for radiation treatment (RT) planning, however, it was noted that this lesion drifted slightly away from the location of the LN. A spot of calcification was noted within this lesion. The CT scan of lower abdomen confirmed that this lesion, size 1.2 × 0.8 cm, was indeed located in the appendix. Considering the high risk of geographic missing of this small mobile lesion, which could result in underdosage and slow response of prostate cancer to RT, this led to changing the treatment to surgical resection. The appendectomy confirmed histological appendiceal metastasis of prostate cancer and presence of appendicolith in the lumen which was dilated distally and contained purulent material. The wall thickness ranged from 0.1 to 0.4 cm. Our case is different from all the previously reported cases in that the disease was solitary, and the diagnosis was made clinically prior to the surgery. This early detection and early surgical treatment likely prevented severe complicated appendicitis associated with prolonged duration of inflammation and appendicolith. The surgical approach avoided adverse effects of radiation to the bowel.

## 1. Introduction

Prostate carcinoma is one of the most common malignancies in men worldwide, second only to lung cancer. According to a recent report by the American Cancer Society, prostate cancer ranks first in incidence among males in 118 of 185 countries, accounting for approximately 66% of all cancer cases worldwide [1].

The metastatic pattern of prostate cancer has been well recognized. Cancer cells usually spread via the lymphatic pathways to pelvic lymph nodes [2] and via valveless vertebral venous plexus to vertebral bones [3], other parts of axial skeleton, and nonaxial bones. Metastasis to the appendix is very rare.

An extensive review of the world English literature disclosed only 11 reported cases of prostate cancer metastasizing to the appendix (Table 1) [4–14]. All except one presented with clinical symptoms of acute appendicitis necessitating surgical management. Many of them were found to have extensive inflammation far beyond the appendix, with some extended to the mesentery. In other cases, adhesion of bowel loops, appendiceal abscess, gangrene, and perforation were present. Most had existing cancer elsewhere such as LN and bone metastasis prior to spreading to the appendix. The only patient who did not have symptoms and did not undergo appendectomy did have existing multiple intra‐abdominal metastasis as well as local recurrence (LR); his diagnosis was based on follow up PSMA PET/CT scan findings.

**Table 1:** World reported cases of metastatic prostate carcinoma to the appendix.

Year	Author	Country	Patient age	Symptoms of acute appendicitis	Existing disease	Significant operative findings
2003	Arya et al. [4]	Iran	70	Yes	Not reported	No report.
2010	Ratanarapee and Nualyong [5]	Thailand	62	Yes	Negative bone scan	Involving periappendiceal tissue appendiceal gangrene.
2013	Ozyazici et al. [6]	Turkey	72	Yes	Bone metastasis	Appendiceal gangrene minor perforation.
2015	El Moussaoui [7]	Belgium	73	Yes	Bone metastasis	Diffuse infiltration of surrounding appendiceal fat.
2016	Lec et al. [8]	United States (Rhode Island)	71	Yes	Biochemical relapses but negative bone scan and abdominal CT scan	Not documented.
2016	Christou [9]	Australia	82	Yes	Bone metastasis	Appendiceal phlegmonous mass adherent to loop of small bowel.
2018	Khan et al. [10]	United States (New York)	72	Yes	Lymph nodes metastasis	Peritoneal deposit in the pelvis.
2020	Campos et al. [11]	Brazil	64	Yes	Bone metastasis	Carcinomatous mass in the mesentery, inflammation of the appendix, caecum, and ascending colon up to hepatic flexure.
2021	Numbere et al. [12]	United States (New York)	66	Yes	None. On hormone treatment.	Dense inflammatory adhesion between bowel loops and the mesentery.
2021	Propst et al. [13]	United States (California)	70	Yes	Lymph nodes metastasis.	Postantibiotics resolution of appendiceal perforation and intra‐abdominal abscess.
2022	Kühl et al. [14]	Denmark	60+	No	Local recurrence, lymph nodes metastasis. Carcinomatosis peritonei.	No appendectomy.

Here, we report a rare case of asymptomatic prostate cancer metastasizing to only the appendix as a solitary disease 17 years after the initial treatment by robotic radical prostatectomy (RP).

## 2. Case Report

A 69‐year‐old man was diagnosed with prostate adenocarcinoma at the age of 52. Cancer involved both lobes. The Gleason score was 7 (4 + 3) in the right lobe and 7 (3 + 4) in the left lobe. His prostate specific antigen (PSA) at time of prostatic biopsy was 6.2 ng/mL. He underwent robotic RP in 2008 (Connecticut, United States). He had been well until June 2022, 14 years after RP when his PSA rose to 2.25 ng/mL, raising suspicion of cancer recurrence. A multiparametric MRI (mpMRI) of the prostate done in July 2022 (Florida, United States) showed a recurrent lesion size of 1.1 × 0.5 cm in the prostatic bed along the left neurovascular bundle near the vesicourethral (VU) anastomosis. A 0.9 cm LN without fatty hilum was also noted at the left anterior perivesical region. Androgen deprivation therapy (ADT) was recommended but the patient declined. In March 2023, his PSA increased to 2.50 ng/mL and further rose to 3.205 ng/mL by early July 2023. The PSMA PET/CT scan (in Florida, United States) showed no distant metastasis. He agreed to and received salvage RT targeting the LR lesion at the left VU anastomosis site during mid‐July to early September 2023. The tumor dose was 73.50 Gy delivered by 35 fractions using volumetric modulated arc therapy technique (VMAT). The metastatic LN at left anterior perivesical region received stereotactic body radiation therapy (SBRT) at 7.2 Gy × 5 fractions.

Following radiation therapy, the PSA decreased from 3.205 ng/mL to 1.34 ng/mL in 3 months, but rose to 1.68 and then to 2.89 ng/mL at 9 months posttreatment. He remained asymptomatic throughout and functioning normally with no urinary complaints or bone pain.

In July 2024, a follow‐up PSMA PET/CT for rising PSA revealed complete resolution of both the irradiated LR lesion at the left UV anastomosis and the metastatic left anterior vesical lymph node. This time a new 0.3 cm lesion was detected on the right side of the lower pelvis adjacent to the levator ani muscle, demonstrating SUVmax of 4.0 and raising the suspicion for recurrence. No other lesions were identified. Given the uncertain nature of this new finding, local RT was not considered but close surveillance was advised. He again declined ADT.

In May 2025, his PSA was slightly increased to 3.22 ng/mL. The PSMA PET/CT scan on August 14, 2025, demonstrated a hypermetabolic lesion, measuring 1.03 cc, SUV max 7.96 at the medial aspect of right common iliac LN region (Figure 1). The previously noted lesion near the right levator ani muscle was no longer present. There was no apparent disease elsewhere. The radiological impression favored metastatic involvement of the common iliac LN from prostate cancer. Salvage SBRT was offered.

**Figure 1:**
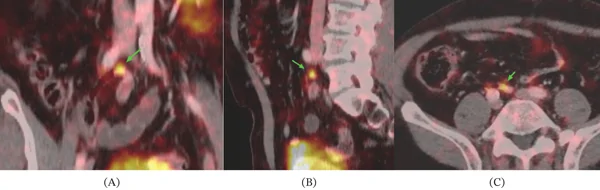
PSMA PET/CT scan demonstrating a hypermetabolic lesion in the right common iliac lymph node region. (A) Coronal. (B) Sagittal. (C) Axial.

On CT simulation for RT planning, it was noted that this lesion appeared to drift away slightly from the LN location, raising suspicion that it was related to the bowel, possibly the appendix. In addition, on careful inspection, there was a small irregular spot of calcification noted within the lesion, possibly a fecalith. To confirm, a contrast‐enhanced CT scan of the lower abdomen was done on August 19, 2025, and it demonstrated a 1.2 × 0.8 cm intensely enhancing nodule at the distal part of the appendix, causing luminal obstruction with resultant dilatation of the appendiceal tip which contained several appendicoliths (Figure 2). This nodular lesion corresponded to the PSMA‐avid focus on the PET/CT scan. However, the small appendicoliths were not clearly distinguishable from adjacent bowel content, probably obscured by the intense adjacent hypermetabolic activity of the tumor. The initial radiologic impression suggested an appendiceal neuroendocrine tumor.

**Figure 2:**
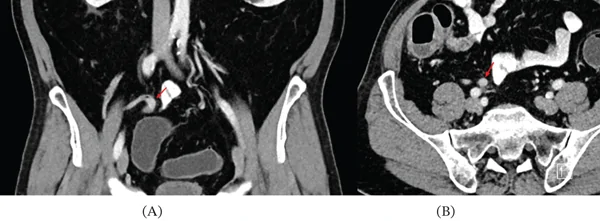
CT scan of the lower abdomen showing enhancing nodule at the tip of the appendix. (A) Coronal. (B) Axial.

The case was discussed in the radiation oncology groups and the surgeon. The treatment was changed from planning of salvage SBRT to surgical resection.

The patient underwent laparoscopic appendectomy on August 26, 2025. Some adhesions from the previous robotic RP performed 17 years ago were noted. There was no peritoneal carcinomatosis seen. The vermiform appendix was found located at the retrocecal position.

Gross pathological examination revealed an appendix measuring 6.5 cm in length and 1.2 cm in maximum diameter, with attached mesoappendix. The serosal surface was dull grayish tan with fibrin deposits. Wall thickness ranged from 0.1 to 0.4 cm. The distal lumen was dilated, contained a small amount of sanguinous and purulent material, as well as an irregular fecalith, without evidence of perforation. Serial sectioning revealed a tan‐white tumor nodule measuring 1.0 × 1.0 × 0.8 cm near the tip, approximately 5 cm from the proximal margin.

Microscopic examination (Figures 3 and 4) confirmed a well‐differentiated adenocarcinoma invading the mucosa, muscularis propria, and subserosal fat (Figures 3 and 4). Perineural and angiolymphatic invasion were seen scattered throughout the appendix to the resection margin. Differential diagnoses included primary appendiceal adenocarcinoma, neuroendocrine tumor and metastatic prostatic adenocarcinoma.

**Figure 3:**
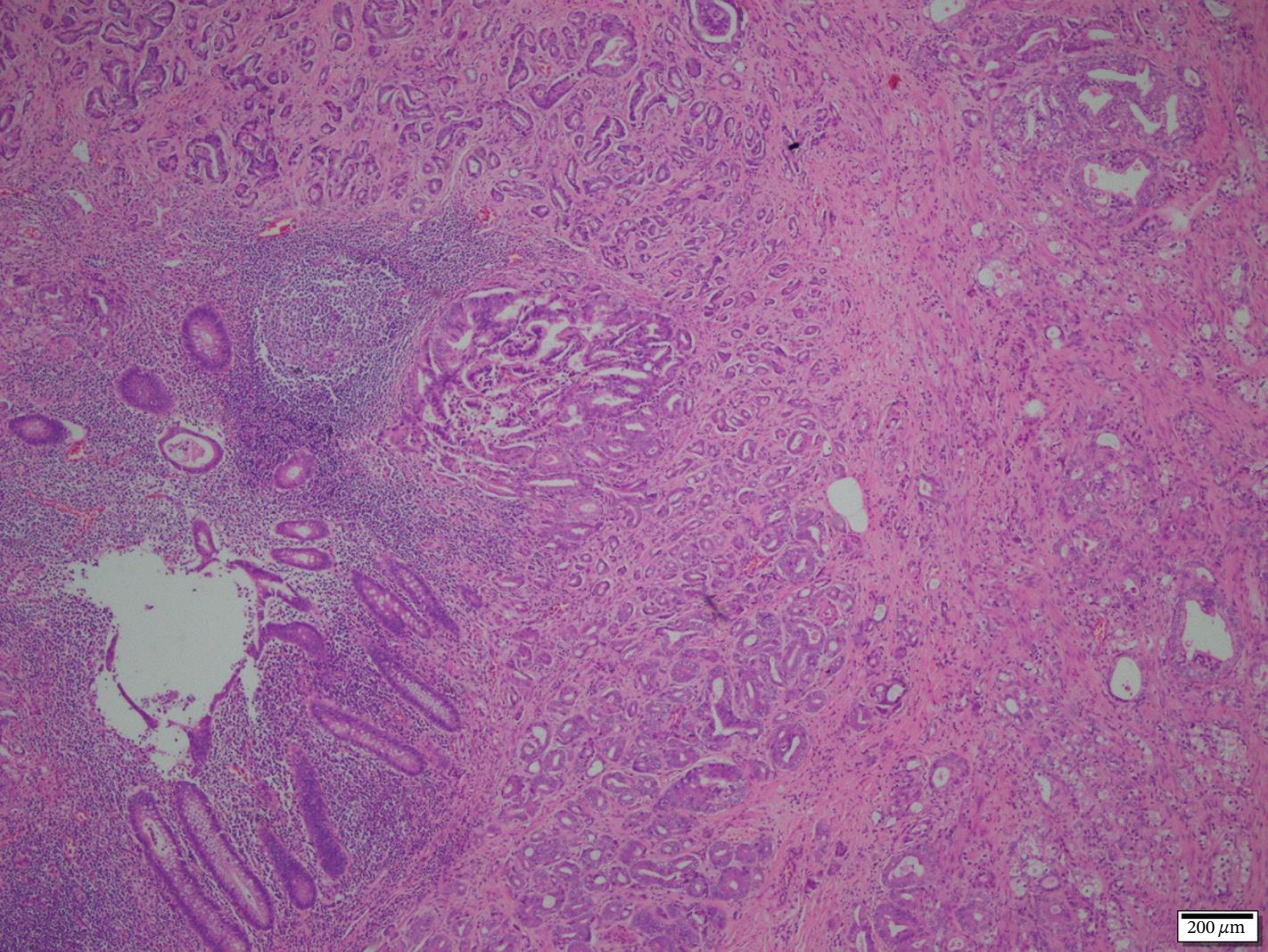
H&E section (original magnification ×40) showing scattered clusters of well‐differentiated neoplastic glands invading the mucosa and muscularis propria.

**Figure 4:**
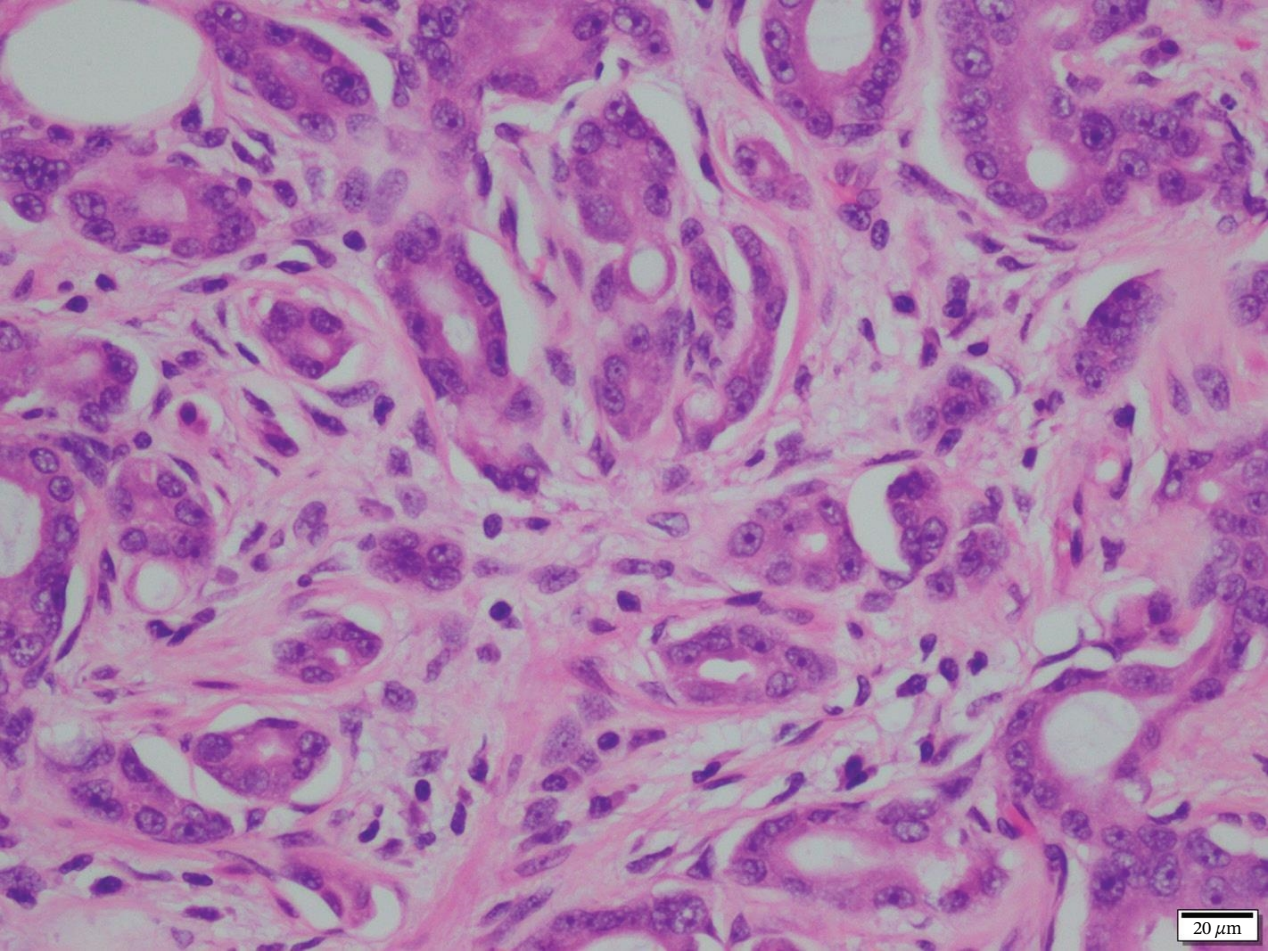
Microscopic view (original magnification ×400) demonstrating a tumor characterized by neoplastic cuboidal cells with round pale‐stained nuclei, prominent nucleoli, and amphophilic cytoplasm arranged in well‐differentiated glands.

Immunohistochemical staining showed tumor cells positive for PSA (cytoplasmic and membranous) and NKX3.1 (nuclear), confirming prostatic origin. Tumor cells were negative for synaptophysin and chromogranin, excluding neuroendocrine tumors, and negative for CK20 and CDX 2, ruling out colorectal adenocarcinoma.

The patient was discharged in stable condition 2 days after surgery. A serum PSA level obtained on the second postoperative day was 3.20 ng/mL; this persistent elevation was not unexpected given the 2‐to‐3‐day half‐life of PSA. ADT was again recommended and again was declined by the patient. At 1‐week follow‐up, he had recovered well from appendectomy and remained asymptomatic from prostate cancer. Shortly thereafter, he returned to his home country with copies of his medical records to ensure continuity of prostate cancer surveillance.

## 3. Discussion

The primary mechanism underlying appendiceal metastasis is likely hematogenous. Because of its small size and relatively low blood flow—unlike highly vascular organs, such as the lungs, and large organs, such as the liver—the appendix is therefore an uncommon site for bloodborne metastasis. Among the primary cancers metastasizing to the appendix causing acute appendicitis, the most common was breast, followed by lung and gastric cancers [15].

Metastasis of prostate cancer to the appendix is exceedingly rare and only 11 other cases were found reported in the world English literature. All but one were diagnosed with the disease postoperatively, after presenting the symptoms of acute appendicitis. The diagnosis of the only case who did not undergo appendectomy was based on follow‐up PSMA PET/CT scan findings. Most patients already had existing cancer elsewhere, suggesting high tumor burden environment, before spreading to the appendix. At time of surgery, several of them were found to have complicated appendicitis, suggesting long duration of inflammatory processes. Two patients, who did not appear to have diseases elsewhere aside from the appendix, did have the basic investigations available at that time (i.e., whole‐body bone scans and CT scans of the abdomen). It was not until 2020 when the high‐sensitivity molecular PSMA PET/CT scan, which can detect the lesion with PSA level as low as 0.5 ng/mL [16, 17], became widely available in medicine. If those two patients were subjected to the PSMA PET/CT scan, then they may be found to have more diseases existing elsewhere, aside from their appendices.

Unlike those 10 symptomatic cases which necessitated appendectomy, our patient did not have the symptoms of acute appendicitis, suggesting that his disease was still in the early stage of inflammation.

Recognition of lesion mobility and noting a spot of calcification within the lesion on CT simulation led to the clinical diagnosis. This case emphasizes the importance of meticulous imaging studies and interpretation, as well as awareness of this unusual metastatic site.

Unlike the previously reported asymptomatic case whose diagnosis was by imaging alone, our patient underwent an appendectomy that histologically confirmed prostate cancer metastasis.

Spot salvage SBRT to this small, mobile target lesion (measuring 1.2 × 0.8 cm) risked geographic miss, both inter‐ and intrafraction, which could result in underdosage. Although RT yielded the same result as the RP in primary prostate cancer treatment, there have been observations that it takes several months to years for PSA levels to reach the nadir. Because of this slow response, coupled with the risk of missing the small mobile lesion, the treatment approach for our patient was thus changed to surgical resection. The appendix was acutely inflamed, dilated, and contained purulent material and appendicolith. With a wall thickness of 0.1 cm, the appendix was at an imminent risk of perforation. Timely surgical removal halted the progression to complicated appendicitis—a severe risk given the presence of an appendicolith, which is highly associated with perforations and postoperative complications [18]. Furthermore, this decisive surgical approach successfully spared the patient′s bowel from unnecessary exposure to radiation.

## 4. Conclusion

We are reporting a new rare case of prostate cancer metastasizing to the appendix.

Our case is different from those 11 previously reported cases in three important aspects. First, the diagnosis was made early and clinically, before the symptoms of acute appendicitis. Second, the diagnosis was not based on imaging alone but confirmed histologically. Third, appendectomy achieved complete removal of the radiologically detectable lesion and possibly prevented appendicolith‐related complications such as perforation and complicated appendicitis, although long‐term oncological cure cannot yet be inferred.

To our knowledge, this represents the first reported case of asymptomatic, histology‐proven solitary appendiceal metastasis from prostate cancer.

## Funding

No funding was received for this manuscript.

## Consent

Consent was given by the patient.

## Conflicts of Interest

The authors declare no conflicts of interest.

## Supporting Information

Additional supporting information can be found online in the Supporting Information section.

## Supporting information


**Supporting Information 1** File S1: Institutional Review Board MedPark Hospital Certificate of Exemption.

**Supporting Information 2** File S2: CARE checklist of information to include when writing a case report.

## Data Availability

Data sharing is not applicable to this article as no datasets were generated or analyzed during the current study.
