# Supplementary material for: Asymptomatic Appendiceal Metastasis From Prostate Cancer Mimicking Metastatic Lymph Node as Solitary Disease on PSMA PET/CT Scan: The First Reported Case of Presurgical Diagnosis and Review of Literature
Source: Case Rep Urol. 2026 Oct 22;2026:7673609. doi: 10.1155/criu/7673609 (PMC13688713; doi:10.1155/criu/7673609)
Supplement: Supplementary file 1 [file CRIU-2026-7673609-s001.pdf]

CARE case report guidelines

CARE Checklist of information to include when writing a case report

(cc) BY-NC-ND

Topic	Item	Checklist item description	Reported on Line
**Title**	1	The diagnosis or intervention of primary focus followed by the words "case report"	2 - 4
**Key Words**	2	2 to 5 key words that identify diagnoses or interventions in this case report, including "case report"	17
**Abstract (no references)**	3a	Introduction: What is unique about this case and what does it add to the scientific literature?	22, 35 - 36
	3b	Main symptoms and/or important clinical findings	25 - 33
	3c	The main diagnoses, therapeutic interventions, and outcomes	33
	3d	Conclusion—What is the main "take-away" lesson(s) from this case?	36 - 38
**Introduction**	4	One or two paragraphs summarizing why this case is unique (**may include references**)	58 - 59
**Patient Information**	5a	De-identified patient specific information.	73
	5b	Primary concerns and symptoms of the patient.	95 - 96
	5c	Medical, family, and psycho-social history including relevant genetic information	N/A
	5d	Relevant past interventions with outcomes	75 - 85
**Clinical Findings**	6	Describe significant physical examination (PE) and important clinical findings.	N/A
**Timeline**	7	Historical and current information from this episode of care organized as a timeline	75 - 85
**Diagnostic Assessment**	8a	Diagnostic testing (such as PE, laboratory testing, imaging, surveys).	95 - 111
	8b	Diagnostic challenges (such as access to testing, financial, or cultural)	N/A
	8c	Diagnosis (including other diagnoses considered)	123 - 130
	8d	Prognosis (such as staging in oncology) where applicable	N/A
**Therapeutic Intervention**	9a	Types of therapeutic intervention (such as pharmacologic, surgical, preventive, self-care)	114
	9b	Administration of therapeutic intervention (such as dosage, strength, duration)	N/A
	9c	Changes in therapeutic intervention (with rationale)	N/A
**Follow-up and Outcomes**	10a	Clinician and patient-assessed outcomes (if available)	140 - 141
	10b	Important follow-up diagnostic and other test results	N/A
	10c	Intervention adherence and tolerability (How was this assessed?)	N/A
	10d	Adverse and unanticipated events	N/A
**Discussion**	11a	A scientific discussion of the strengths AND limitations associated with this case report	162 - 164
	11b	Discussion of the relevant medical literature **with references**.	69 - 71, 149 - 151
	11c	The scientific rationale for any conclusions (including assessment of possible causes)	162 - 164
	11d	The primary "take-away" lessons of this case report (without references) in a one paragraph conclusion	162 - 164
**Patient Perspective**	12	The patient should share their perspective in one to two paragraphs on the treatment(s) they received	N/A
**Informed Consent**	13	Did the patient give informed consent? Please provide if requested	**Yes** ☑ **No** ☐
