# Supplementary material for: Asymptomatic Appendiceal Metastasis From Prostate Cancer Mimicking Metastatic Lymph Node as Solitary Disease on PSMA PET/CT Scan: The First Reported Case of Presurgical Diagnosis and Review of Literature
Source: Case Rep Urol. 2026 Oct 22;2026:7673609. doi: 10.1155/criu/7673609 (PMC13688713; doi:10.1155/criu/7673609)
Supplement: Supplementary file 2 [file CRIU-2026-7673609-s002.pdf]

3333 Rama IV Road, Khlong Toei, Bangkok
10110 Tel. 02-023-3333

COE: 002/2026

MedPark Hospital

INSTITUTIONAL REVIEW BOARD

MEDPARK HOSPITAL

CERTIFICATE OF EXEMPTION

Project Title: Asymptomatic Appendiceal Metastasis from Prostate Cancer Mimicking Metastatic Lymph Node as solitary disease on PSMA PET/CT scan: The First Reported Case and Review of Literature.

Principal Investigator: Asst. Prof. Dr. Sunantha Srisubut-Ploysongsang

Date of Meeting: -

Date of Acknowledgement: 23 June 2026

The Institutional Review Board of the MedPark Hospital has exempted the following study in compliance with the international guidelines for human research protection as Declaration of Helsinki, The Belmont Report, CIOMS Guideline, International Conference on Harmonization in Good Clinical Practice (ICH-GCP) and 45CFR 46.101(b)

Signature..

(Professor Dr. Siriwan Tangjitagmol)

Chairman of

MedPark Institutional Review Board

MedPark IRB
APPROVED 23/06/26

All approved investigators must comply with the following conditions: Specify investigator (s) to report the committee if there is a change in the protocol (amendment) and send the final report after finishing and publication of the research.

MedPark Hospital<br>**Medical Certificate (General Purpose)**	**Name** : Mr. Tony Ferris Hadeed<br>**Date of Birth**: 02/08/1956 **Sex** : Male **Age** : 69yrs<br>**HN** : 689020832 **Date** : 03/09/2025<br>**Physician** : Asst. Prof. Dr. Sunantha Srisubut-Ploysongsang<br>**Allergy** : No known allergies

สถานที่ตรวจ : โรงพยาบาลเมดพาร์ค
Place of examination : MedPark hospital
สถานที่ประกอบวิชาชีพเวชกรรม : 3333, ถนนพระรามที่ 4 คลองเตย กรุงเทพมหานคร 10110 ประเทศไทย
Practicing medicine at 3333, Rama IV Rd, Klong Toey, Bangkok 10110 Thailand
วันที่รับการตรวจ / Date of Examination : 03/09/2025

ผลการตรวจประเมินวินิจฉัยโรค
Assessment :

Known case of Prostate cancer treated by Robotic radical prostatectomy in year 2008 (Connecticut , USA) . Cancer relapse after 14 years at left side of urethrovesical anastomosis and at lymph node at anteroperivesical region. These lesions were treated successfully by radiation therapy(July - Sept. 2023) In year 2025 the PSA became elevated to 3.2 ng/mL. and PSMA PET / CT scan (Aug. 14 ,2025) showed a hypermetabolic lesion at common iliac region.The impression was metastatic disease to the lymph node. No apparent disease elsewhere.The treatment recommendation was radiation therapy. CT simulation showed this lesion was moved away from common iliac region and looked like an appendiceal tip rather than the lymph node. The CT of lower abdomen with intravenous contrast and oral bowel and rectum contrast (Aug. 19 ,2025) confirmed the lesion located at the distal end of appendix.The patient underwent Laparoscopic appendectomy on Aug. 28 ,2025 . The pathology report the appendix size 6.5 cm. in length and tip 1.2 cm in width. Microscopically this was well differentiated adenocarcinoma involving the mucosa , muscular layer and subserosa fat . There are perineural and angiolymphatic invasion. IHC studies ruled out colonic cancer and neuroendocrine tumor but positive for Prostate adenocarcinoma.

สรุปความคิดเห็น และ/หรือ ข้อแนะนำของแพทย์
Summary and/or Recommendation :

The appendiceal metastasis from prostate cancer is very rare and approximately only 10-12 cases were reported in the medical literature.Therefore we would like to report this case so that the physicians would be aware of this unusual behavior of prostate cancer.
There will be no mention of the name or identification of the patient in the report.
The patient agreed with this action for medical education purpose.

ขอรับรองว่าข้อความข้างต้นเป็นความจริง
I certify that the above statements are true

MedPark Hospital

(ลงชื่อ)..แพทย์ผู้ตรวจ
Asst. Prof. Dr. Sunantha Srisubut-Ploysongsang
Physician's Signature
ใบประกอบวิชาชีพเวชกรรม เลขที่ / License No. 4449

(ลงชื่อ)..ผู้รับการตรวจ
Mr. Tony Ferris Hadeed
Patient's Signature
